# Thermal Comfort Index for Lactating Water Buffaloes under Hot and Humid Climate

**DOI:** 10.3390/ani11072067

**Published:** 2021-07-11

**Authors:** Mengwei Li, Xin Liang, Zhenhua Tang, Faiz-ul Hassan, Lili Li, Yanxia Guo, Kaiping Peng, Xianwei Liang, Chengjian Yang

**Affiliations:** 1 Key Laboratory of Buffalo Genetics, Breeding and Reproduction Technology, Ministry of Agriculture and Guangxi Buffalo Research Institute, Chinese Academy of Agricultural Sciences, Nanning 530001, China; lmw1607@163.com (M.L.); liangxinbri@163.com (X.L.); tangzhyaya@163.com (Z.T.); f.hassan@uaf.edu.pk (F.-u.H.); lily799802@163.com (L.L.); gyxlq0417@163.com (Y.G.); pkp900927@163.com (K.P.); liangbri@126.com (X.L.); 2 Institute of Animal and Dairy Sciences, Faculty of Animal Husbandry, University of Agriculture, Faisalabad 38040, Pakistan

**Keywords:** buffalo, thermal comfort, physiological parameters, heat stress

## Abstract

**Simple Summary:**

Heat stress drastically affects the productive and reproductive performance of animals in addition to causing welfare issues. Therefore, thermal comfort is an important consideration to avoid performance losses and other adverse effects of heat stress on animal physiology under various production systems. Moreover, it is becoming more important under the recent scenario of climate change. The present study was conducted to develop a thermal comfort index for buffaloes. Physiological parameters of buffaloes and environmental variables were recorded to develop the index models through typical correlation. The most accurate model was based on body surface temperature, rectal temperature and respiratory rate and can be used effectively to indicate the state of thermal comfort in buffaloes under hot and humid climate.

**Abstract:**

Heat stress results in serious performance losses and adversely affects animal health and welfare under various production systems. This study was conducted to develop a thermal comfort model for lactating buffaloes under hot and humid climate. Twenty Nili-Ravi buffaloes were randomly enrolled for this one-year study. Physiological parameters including rectal temperature (RT), respiratory rate (RR), and body surface temperature (BST) and environmental variables such as wet bulb temperature (WBT), dew point temperature (DPT), and black globe temperature (BGT) were recorded twice a week on each Tuesday and Thursday (*n* = 1602 and 1560, respectively) at 8:00 am and 2:30 pm. Moreover, ambient temperature (AT, °C) and relative humidity (RH, %), at an interval of every 30 min were recorded. We used a typical correlation analysis to build the index models for thermal comfort. The results revealed that AT positively correlated with BGT, WBT, DPT, BST, RT, and RR, while RH negatively correlated with RT. Moreover, a physiological index model consisting of BST, RT and RR (P1 = 0.578 × BST + 0.047 × RT + 0.429 × RR) and an environmental index model (E1 = 0.881 × AT + 0.194 × RH + 0.455 × BGT − 0.347 × WBT + 0.032 × DPT) proved to be a more accurate index as a pair to reveal the state of thermal comfort in lactating buffaloes. Moreover, these models correlated well with physiological variables, indicating that this this pair of index models can be used to effectively evaluate the thermal comfort in buffaloes.

## 1. Introduction

Animals have been intensively selected for breeding to increase performance to address ever increasing demand for food products and input costs. However, climate changes are gradually resulting in global warming which has negative effects on the performance and health of high producers [1]. Analysis of global meteorological data of the last 50 years has indicated an increase of 0.13 °C/year in the global temperature [2]. High-temperature episodes for a longer time adversely affect milk yield and quality leading to huge economic losses to the dairy industry [3]. Furthermore, higher relative humidity along with high temperatures can aggravate this situation by substantially increasing the temperature-humidity index (THI). Although buffaloes can adapt quite well to relatively hot environmental conditions, dark body-color [4], less number of sweat glands [5], and low hair density [6], make buffaloes more susceptible to thermal radiation leading to heat stress and endocrine imbalances than zebu cattle [7]. Despite the well-developed thermoregulatory system, exposure of buffaloes to higher ambient temperatures (≥36°C) adversely affects their performance [8,9,10]. Moreover, high milk production and exposure to hot and humid climate especially under lack of proper shelter, wallowing, and/or swimming provisions, increases the susceptibility to heat stress in buffaloes [11]. Higher metabolic rate and heat generated during rumen fermentation make dairy animals (e.g., buffaloes) more prone to heat stress, because milk yield is associated with high metabolic heat production in the body [12].

In-depth monitoring of the thermoregulatory response of buffalo under subtropical climate conditions is essentially required to avoid adverse effects of environmental factors on buffalo production [13,14]. High environmental temperature coupled with high humidity have been shown to adversely affect growth, reproduction and production in buffaloes, in addition to creating welfare issues [8,10]. The thermal comfort index (TCI), usually used to investigate animal comfort, is a general index as akin to the THI index, which cannot be universally applied to buffaloes raised in different areas. Furthermore, a major buffalo production system involves housing of buffaloes in sheds and stall feeding, which requires regular monitoring of thermal comfort as the animal shed is the main place for buffalo activities. In this regard, continuous monitoring of environmental variables in an animal shed is inevitable to elucidate the level of thermal comfort. We hypothesized that owing to different physiology and thermoregulation in buffaloes as compared to cattle, level of thermal comfort varies in this species under different sets of environmental conditions. Therefore, a specific thermal comfort index model is required for buffaloes for the elucidation of the thermal comfort of animals under captivity to monitor and address heat stress challenges. This study aimed to develop a model for evaluating the thermal comfort state in buffaloes using physiological and environmental parameters under hot and humid climate.

## 2. Materials and Methods

### 2.1. Study Location and Agro-Climatic Conditions

The present study was conducted for one year from April 2017 to March 2018 at the buffalo breeding farm of Guangxi Buffalo Research Institute in Nanning city of Guangxi province (22°53′22.59″ N and 108°21′51.19″ E; altitude 122 m). The real-time recording of AT and RH was performed daily while physiological parameters and environmental parameters (BGT, WBT, DPT) were recorded weekly twice on each Tuesday and Thursday. The annual ambient temperature of the study area ranged from −2.4 °C to 40.4 °C with an average of 21.6 °C. The coldest month in winter had an average temperature of 12.8 °C, while the hottest month in summer had an average of 28.2 °C. The average relative humidity was 79% throughout the year. Generally, the weather in this area is usually wet in summer, but slightly dry in winter (Guangxi Meteorological Bureau, http://gx.cma.gov.cn/, accessed on 1 May 2018).

### 2.2. Animal Management and Housing

Twenty Nili-Ravi lactating buffaloes 5–8 years old (parity 3 ± 1) were selected for this study. The average body weight of the selected animals was 575 ± 23 kg, with an average milk yield of 6.13 ± 0.58 kg/d (having average days in milk of 90 ± 10 days). All buffaloes were fed twice a day (6:00 AM and 2:00 PM) with an allowance of total mix ration (TMR) mainly consisting of grass (*Pennisetum purpureum schum*), brewer’s grain, cassava residue, and maize. Detailed formulation and composition of the TMR is given in Table S1. For round the clock water availability, there were two water troughs at an open area measuring 1 m (width) × 3 m (length) × 1 m (height), full of water every time.

Buffaloes were housed in the animal shed during the milking time while for the rest of the time, they were set free in an open area. An open exercise area was provided with a density of about 15 m^2^/head. Recording of physiological variables was performed in the milking parlor. The animal shed was equipped with the facilities of water spray and electric fans for better water sprinkling and airflow, respectively. The cows were housed in the pens for milking at 6:00 AM–9.00 AM and 2:00 PM–5.00 PM. Before each milking, buffaloes were allowed to swim for 30 min in a water pond. After swimming, buffaloes were sprayed with tap water to clean their bodies in the shed.

### 2.3. Recording of Meteorological Data

We used an online dust monitoring system (Shenzhen Greenforze Environmental Technology Co., Ltd. Shenzhen, China) to record daily meteorological data in real-time, mainly including air temperature (AT, °C) and relative humidity (RH, %), at an interval of every 30 min. The device was installed at a height equal to the height of the animal’s back and can measure a temperature range from −20~80 °C with a precision value of ±0.5 °C (error rate ±1%).

Manual recording of wet bulb temperature (WBT, °C), black bulb temperature (BLB, °C), and dew point temperature (DPT, °C) was performed in the morning and afternoon twice a week on each Tuesday and Thursday by using the black globe device (AZ8758, Shenzhen Yuanhengtong Technology Co., Ltd. Shenzhen, China), which can record between approximately 0 and 50 °C with a precision value of ±0.1 °C. We recorded data twice per week as the data recorded on Tuesday were used to develop the index model, while the data collected on Thursday was used for the verification of the model’s applicability as reported previously [15].

### 2.4. Measurement of Physiological Parameters of Buffalo

Recording of physiological parameters was performed in the morning and afternoon twice a week on each Tuesday and Thursday for one year. The rectal temperature (RT, °C), body surface temperature (BST, °C), and respiratory rate (RR, breaths/min) of buffaloes were recorded in the shed in the morning between 8:00 and 9:00 AM and in the afternoon between 2:30 and 3:30 PM). The rectal temperature was recorded by an animal rectal thermometer (GLA 700, GLA Corporation, San Luis Obispo CA 93401, U.S), and the shortest maximum temperature was taken as the rectal temperature value by keeping the thermometer in the rectum for two minutes. Body surface temperature was detected by an animal infrared thermometer (HRQ-S60, Zhengzhou Haorunqi Electronic Technology Co., Ltd., Zhengzhou, Henan, China), and the average temperature value of three body sites (forehead, left chest, and left abdomen) was taken as the surface temperature of the buffalo.

Respiratory rate (breathes/minute) was recorded by manual counting method using stopwatch through observation of chest and abdomen movements for 2 min, and we calculated the average value of 1 min as the RR.

### 2.5. Development of Thermal Comfort Index

The comfort level of buffalo was categorized into comfortable, critical, stress, and dangerous states based on the observation of three physiological indicators. The reference values used for each physiological parameter of this index as reported previously [4] were: rectal temperature from 37.4 to 37.9 °C, body surface temperature from 25.6 to 35.5 °C, respiratory rate 18 to 30 breaths/min.

A total of 1602 observations for the three physiological parameters (RT, BST, and RR) collected on Tuesday were used to determine the thermal comfort index model by the typical correlation analysis. Another set of 1560 observations for the same parameters collected on Thursday were used to verify the reliability of the developed equation by using newly developed models. Thermal comfort equations assumed for this study were as follows:

E (Environmental index model)=a(AT)+b(RH)+c(BGT)+d(WBT)+e(DPT)

P(Physiological index model)=a(BST)+b(RT)+c(RR)

In the formula, *E* is used to represent the climate variables, and the buffalo comfort climate condition index is determined. The *P* is used to represent the buffalo physiological variable, and the buffalo comfort physiological index is determined. The correlation coefficient is *R*. The animal’s comfort range is determined by the status defined as the mean (M) and the standard deviation (SD) of the index, as shown in Table 1 [15].

A comparison of newly developed index models was made with the existing models which have been widely used to evaluate the state of heat stress in animals (Table 2).

### 2.6. Statistical Analysis

The typical correlation analysis was used to determine the impact of climate variables (AT, RH, WBT, BGT, DPT) on physiological variables (BST, RR, RT) using the SPSS software (SPSS 19.0, 2014). The chi-square test (*p* < 0.05) was used to verify the validity of the model. The conversion formula of canonical correlation variables between the two types of factors was obtained, and the retained canonical correlation coefficient was determined according to the proportion of the variance of original variable explained by each canonical correlation coefficient, to develop the index model. Pearson’s correlation analysis was used to determine the association of previously reported models (THI, GTHI, BICT, and ITC) with our newly developed index as well as with physiological (RT, BST and RR) and climatic variables (AT, RH, DPT, WBT, and BGT). The significance was considered at *p*-values less than 0.05 (*) and 0.01 (**).

## 3. Results

The results of the present study revealed the correlation among environmental variables and physiological parameters of buffaloes (Table 3). The typical correlation analysis of environmental variables including AT, RH, BGT, and DPT with physiological parameters including BST, RT, and RR is presented in Table 4. Results revealed the first typical correlation coefficient to be the largest and statistically significant (*p* = 0.001), indicating a strong association between both indicators. Therefore, the environment variables and the buffalo physiological variables can be mutually derived from the following index models (general model):

E1 (Environmental index model)=0.881AT+(0.194RH)+(0.455BGT−0.347WBT)+0.032DPT)

P1(Physiological index model)=0.578BST+0.047RT+0.429RR

The typical correlation analysis effective model with three environmental variables including AT, RH, and BGT, and three physiological indicators including BST, RT, and RR is presented in Table 5. The first typical correlation coefficient was maximum, with a *p* value = 0.001 indicating a close association between both indicators. Therefore, the environment variables and the buffalo physiological indicators can be mutually derived from this index model (effective model) as follows:

E2 (Environmental index model)=0.602AT+0.137RH+0.421BGT

P2 (Physiological index model)=0.584BST+0.048RT+0.421RR

The typical correlation of the third model was based on two environmental variables (AT and RH) and two physiological indicators (BST and RR) (Table 6). The first typical correlation coefficient was the largest, with a *p*-value of 0.001 indicating a strong correlation between the two types of indicators. Therefore, the environment variables and the buffalo physiological index variables can be derived from this index model (practical model) as follows:

E3 (Environmental index model)=1.016AT+0.139RH 

*P3 (physiological index model) = 0.654 BST + 0.381 RR*

According to the definition of animal comfort stated in Table 1, the state of the buffaloes under the new index model was analyzed (Table 5). The status classification of lactating buffaloes under the environmental index model (*E*) is presented in Table 6. Three environmental index models showed a good agreement, and the consistency of the effective model and practical model with the general model was 87.69% and 85.45%, respectively. The classification of lactating buffalo states under the physiological index model (*P*) is presented in Table 7.

Three physiological index models also showed good agreement, and the consistency of the effective model and practical model with the general model was 96.15% and 98.14%, respectively. The consistency of environment and physiological index models for lactating buffaloes were summarized in Table 8. 

The correlation between environmental indices (THI, GTHI, E1, E2, E3) and physiological indicators (BST, RT, RR) of buffaloes is presented in Table 9. The correlation between physiological indices (BTCI, IHTI, P1, P2, P3) and physiological indicators (BST, RT, RR) is shown in Table 10. All index models showed a significant correlation with physiological indicators of buffaloes. The new models showed that it can be safely used to indicate environments that are likely to cause thermal stress in buffaloes. 

## 4. Discussion

Physiological indicators are the most intuitive indicators of heat stress in the buffalo. Under normal circumstances, the physiological indices of the buffalo remain relatively constant. Rectal temperature is the most representative and practical indicator in animals [20]. Skin is the main site for heat exchange between the surface of the mammal and the environment. The BST is manifested by the regulation of skin blood flow after heat exchange between the inner core of the animal body and the skin [21].

In the present study, we observed a strong correlation between BST and RT (0.65) in buffalo, which is consistent with earlier reports [22]. Moreover, a positive correlation between ambient temperature and eardrum temperature has also been reported [23]. Our findings also confirmed that RT, AT, and WBT were positively correlated with each other. The RR of buffaloes can be used as a reference for various diseases, such as compensatory acidosis. An increase in body temperature is usually accompanied by an increase in RR [24]. Our study showed a positive correlation of RT with RR. The BST has also been positively correlated with RR so it can also be used as an indicator to reflect environmental conditions [25]. In our study, both BST and AT were positively correlated with RR.

The thermal environment is a collective manifestation of the effects of temperature, humidity, wind speed, and other factors. A single indicator cannot fully reflect the thermal environment of the buffalo house. The AT is the main thermal factor that affects the health and production performance of animals. The temperature inside the barn is mainly deter-mined by its own heat production, solar radiation, and ventilation. Air humidity affects the evaporation of water on the body surface of the buffalo leading to reduced body heat dissipation at higher humidity levels. Because temperature and humidity have an interactive effect on animals, the THI is generally used to evaluate the thermal environment. However, one study has reported RH, AT, and BGT as the most important factors of thermal environment, which can collectively indicate the overall effects of temperature, relative humidity, solar radiation, and airflow on animals [26]. Therefore, compared to the general index models (E1 and P1), the effective index models (E2 and P2) are relatively easier to record and reflect the thermal comfort of buffalo. However, it is impossible to judge the physiological state of buffaloes only from meteorological data. However, our findings revealed that a physiological index model consisting of BST, RT and RR (P1) and an environmental index model (E1) proved to be a more accurate index as a pair to reveal the state of thermal comfort in lactating buffaloes. No doubt E1 and P1 are relatively more accurate, but they are also time and labor demanding as they require measuring different variables. Therefore, practical index models (E3 and P3) are much better to feasibly obtain environment (AT and RH) and physiological data (BST and RR) without causing additional stress to buffaloes and are also suitable for quickly obtaining the thermal comfort state of buffaloes.

General, effective, and practical models can be used for the evaluation of climatic conditions of a buffalo house and define thermal comfort in lactating buffaloes. The consistency between the general environmental model, the effective model, and the practical model was 87.69% and 85.45%, respectively, indicating that both the environmentally effective and practical index model could be used as a basis for evaluating the state of heat stress in the buffalo. The consistency between the general physiological model, the effective model, and the practical model was 96.15% and 98.14%, respectively, indicating that the physiologically effective and practical index model is equally effective to evaluate the thermal comfort in buffalo, which is consistent with previous reports indicating that different index models can be used to assess comfort level in buffaloes [15]. 

The thermal humidity index is an index that uses temperature and humidity to comprehensively assess the degree of heat. It can more precisely reflect the degree of environmental stress on dairy cows than simple temperature indicators [27]. Our findings regarding the positive association of GTHI with RT, BST, and RR of buffaloes is in agreement with earlier studies [28,29].

No doubt already existing environmental index models showed significant association with buffalo physiological indicators, but the newly developed model in the present study had the highest correlations especially with BST. Earlier studies have also reported a strong negative correlation between IHTI and RT in dairy cows in different seasons in the tropics [30]. On the other hand, studies have shown that BTCI was positively correlated with mean air temperature, which indicates thermal stress at higher temperatures [31]. Three physiological index models obtained in the present study were positively correlated with buffalo physiological indicators, especially with BST and RR. The strong correlation between the environmental index model and the physiological index model and the buffalo physiological parameters indicates that the new index model developed in this study can be applied effectively to evaluate the states of thermal comfort in lactating buffaloes under the hot and humid climate. Studies have reported that a model related to sensible heat (ambient temperature-humidity) possesses similar values to those of other THI models, but is more effective at higher values of humidity, suggesting superiority of the respective model developed in this study for the detection of thermal stress under hot and humid conditions [32].

## 5. Conclusions

Our findings indicated significant correlations between the physiological indexes of buffaloes and the environmental indicators of the shed. The new index models and the physiological indices of lactating buffaloes were significantly correlated. Therefore, index models developed in the present study are all equally applicable for the evaluation of thermal comfort in buffaloes raised in subtropical areas. In particular, the practical index model is more feasible and easier to use during lactation, thus providing a scientific tool for effective regulation of microclimate in a buffalo production system.

## Figures and Tables

**Table 1:** Definition of range of animal comfort.

Animal Status	Range
Comfort	≤M
Danger	M~M + SD
Stress	M + SD~M+2 × SD
Emergency	≥M+2 × SD

**Table 2:** Previously reported thermal comfort models used for comparison with new models.

S. No	Name of the Model	Formula	Reference
1	Thermal humidity index (THI)	THI = AT + 0.36 DPT + 41.5	[16]
2	Black ball temperature and humidity index (GTHI)	GTHI = BGT + 0.36 DPT + 41.5	[17]
3	Benezra’s thermal comfort index (BTCI)	BTCI = (RT/38.8) + (RR/23)	[18]
4	Rhoad’s heat resistance index (IHTI)	IHTI = 100 − 18 (RT − 38.33)	[19]

**Table 3:** Pearson’s correlation analysis of environmental and physiological indices of lactating buffaloes.

Items	AT	RH	BGT	WBT	DPT	BST	RT	RR
AT	1							
RH	−0.1799	1						
BGT	0.9748 **	−0.2064	1					
WBT	0.9566 **	0.0064	0.9311 **	1				
DPT	0.8962 **	0.1071	0.8660 **	0.9846 **	1			
BST	0.9213 **	−0.0657	0.9064 **	0.8888 **	0.8319 **	1		
RT	0.6614 **	−0.3956 **	0.6291 **	0.5741 **	0.5082 **	0.6517 **	1	
RR	0.8749 **	0.0043	0.8807 **	0.8523 **	0.8084 **	0.8554 **	0.5033 **	1

** *p* value < 0.01.

**Table 4:** Analysis of typical correlation coefficients between environmental variables of buffalo house and physiological indicators of lactating buffaloes (general model).

Model	Typical Correlation Coefficient	Typical Correlation Coefficient Squared	Chi-SQ	Degrees of Freedom	*p* Value
General	0.9520	0.9063	197.814	15.00	0.001
	0.5190	0.2693	28.044	8.00	0.001
	0.2740	0.0750	5.573	3.00	0.134
Effective	0.9500	0.9025	192.764	9.00	0.001
	0.5190	0.2693	23.797	4.00	0.001
	0.1210	0.0146	1.076	1.00	0.300
Practical	0.9450	0.8930	165.623	4.00	0.001
	0.1270	0.0161	1.204	1.00	0.273

**Table 5:** Response scale of the developed comfort index of buffaloes.

Exponential Model	Mean ± SD	Comfort	Danger	Stress	Emergency
*E1*	42.65 ± 7.29	≤42.65	42.65~49.94	49.94~57.23	≥57.23
*P1*	25.47 ± 3.27	≤25.47	25.47~28.74	28.74~32.01	≥32.01
*E2*	37.07 ± 6.97	≤37.07	37.07~44.04	44.04~51.01	≥51.01
*P2*	25.57 ± 3.26	≤25.57	25.57~28.83	28.83~32.09	≥32.09
*E3*	37.15 ± 6.91	≤37.15	37.15~44.06	44.06~50.97	≥50.97
*P3*	25.30 ± 3.34	≤25.30	25.30~28.64	28.64~31.98	≥31.98

E effective model, P physiological model.

**Table 6:** Relationship among the classification of different environmental comfort indices.

General Model	Effective Model				Practical Model				Total (Head)
	Comfort	Danger	Stress	Emergency	Comfort	Danger	Stress	Emergency	
Comfort	812	48			812	48			860
Danger	74	291	6		89	266	16		371
Stress		58	244	3		71	234	3	308
Emergency				21				21	21
Total (head)	886	397	250	24	901	385	250	24	1560
Consistency	87.69% (1368/1560)				85.45% (1333/1560)				

**Table 7:** Relationship among the classification of different thermal comfort models.

General Model	Effective Model				Practical Model				Total (Head)
	Comfort	Danger	Stress	Emergency	Comfort	Danger	Stress	Emergency	
Comfort	844	25			852	17			869
Danger		320	21		4	330	7		341
Stress		14	274				287	1	288
Emergency				62				62	62
Total (head)	844	359	295	62	856	347	294	63	1560
Consistency	96.15% (1500/1560)				98.14% (1531/1560)				

**Table 8:** Comparison of consistency between environmental and physiological index models.

Animal Status	General Model	Effective Model	Practical Model
Comfort	760	728	721
Danger	264	209	186
Stress	129	135	108
Emergency	7	7	7
Total (head)	1160	1079	1022
Consistency	74.36%	69.17%	65.51%

**Table 9:** Correlation analysis of environmental indices and physiological indicators of buffaloes.

Category	THI	GTHI	E1	E2	E3
BST	0.916 **	0.911 **	0.898 **	0.911 **	0.906 **
RT	0.634 **	0.614 **	0.503 **	0.535 **	0.539 **
RR	0.875 **	0.885 **	0.888 **	0.894 **	0.882 **

** means significantly correlated at *p* < 0.01.

**Table 10:** Correlation analysis of physiological indices and physiological indicators of buffaloes.

Category	BTCI	IHTI	P1	P2	P3
BST	0.861 **	−0.652 **	0.970 **	0.971 **	0.977 **
RT	0.528 **	−1.000 **	0.609 **	0.610 **	0.615 **
RR	0.999 **	−0.504 **	0.956 **	0.954 **	0.946 **

** means significantly correlated at *p* < 0.01.

## Data Availability

No data were generated or analyzed in this study.

## Supplementary Materials

The following are available online at https://www.mdpi.com/article/10.3390/ani11072067/s1, Table S1: Formulation and chemical composition of total mix ration fed to lactating buffaloes (on air-dry basis).
