# Supplementary material for: Thermal Comfort Index for Lactating Water Buffaloes under Hot and Humid Climate
Source: Animals (Basel). 2021 Jul 11;11(7):2067. doi: 10.3390/ani11072067 (PMC8300202; doi:10.3390/ani11072067)
Supplement: Supplementary file 1 [file animals-11-02067-s001.zip › animals-1280327-supplementary.pdf]

Supplementary Table

Table S1

Table S1 Formulation and chemical composition of Total Mix Ration fed to lactating buffaloes (% on air-dry basis)

**Items**	**Content %**
Grass (*Pennisetum purpureum schum*)	12
Brewer's grain	21
Cassava residue	33
Corn	17.83
Wheat bran	7.51
Soybean meal	5.72
Lime stone	0.5
$CaHPO_4$	0.6
$NaHCO_3$	0.8
NaCl	0.7
Vitamin-Mineral Premix[1)]	0.34
Total	100
Nutrient levels[2)]	
CP	14.6
NDF	36.21
ADF	23.5
Ash	6.23

[1]The additive premix provided the following per Kg of diets: Vit. A 550 000IU,Vit. E 3000 IU, Vit. D3 150 000IU, Fe (as ferrous sulfate) 4.0g, Cu (as copper sulfate) 1.3g, Mn (as manganese sulfate) 3.0g, Zn (as zinc sulfate)6.0g, Co(as cobalt sulfate) 80mg.

[2]Measured values
